# Comparison of clinical characteristics and outcomes among different age groups in elderly patients with hip fracture surgery

**DOI:** 10.1371/journal.pone.0333909

**Published:** 2025-10-15

**Authors:** Qingwen Cao, Lize Zhang, Xingzhi Cai, Wenlong Shen, Haoran Wu, Ailan Yu, Xia Xu

**Affiliations:** 1 School of Anesthesiology, Binzhou Medical University, Binzhou, Shandong, China; 2 Department of Anesthesiology, Liaocheng People’s Hospital, Liaocheng, Shandong, China; 3 Department of Anesthesiology, Zibo Central Hospital, Zibo, Shandong, China; 4 Department of Anesthesiology, The Fifth People’s Hospital of Jinan, Jinan, Shandong, China; Southern Medical University Nanfang Hospital, CHINA

## Abstract

**Background:**

Studies comparing the clinical characteristics and outcomes among different age groups in elderly patients with hip fractures are scarce. Thus, this study aimed to 1) analyze the clinical characteristics and postoperative outcomes among different age groups in elderly patients undergoing fracture surgery. 2) provide more evidence-based basis for perioperative management.

**Methods:**

In the retrospective study, 909 patients aged 65 years and above who underwent hip fracture surgery at Liaocheng People’s Hospital between October 2020 and April 2023 were categorized into three groups based on age: young old group (YO group, 65–74 years old), middle old group (MO group, 75–84 years old), and oldest-old group (OO group, ≥ 85 years old). Then, their pre-fracture conditions, fracture causes and type, anesthesia and surgical details, postoperative complication and outcomes were compared.

**Results:**

In the YO, MO, and OO groups, the male-to-female ratios were 1:2.1, 1:1.8, and 1:2.3, respectively (**P* *=* *0.293). The proportion of patients with American Society of Anesthesiologists (ASA) ≥ III, Nottingham hip fracture score (NHFS) ≥ 5 and preoperative activity tolerance < 4 metabolic equivalents (METs) increased sequentially (**P* *< 0.001), across the YO, MO, and OO groups, whereas the proportion of those with three or more internal medicine comorbidities sequentially decreased (**P* *< 0.001). All three groups were primarily injured by falls and the older the age, the higher the proportion of injuries caused by falls (**P* *= 0.006). There was a higher proportion of femoral neck fractures in the YO group and a higher incidence of intertrochanteric fracture in the OO group (**P* *<** 0.001). The proportion of patients undergoing spinal anesthesia in the HO group (78.8%) was higher than that in the MO (59.9%) and YO groups (55.1%) (*P* < 0.001). Additionally, the proportion of postoperative intensive care unit (ICU) admissions (10%) was higher in the OO group compared to the MO (6.2%) and YO groups (4.5%) (**P* *= 0.032). Meanwhile, the incidence of postoperative complications progressively increased in the YO, MO, and OO groups (*P* < 0.001). The mortality rates of the HO group at the hospital, 30 days postoperatively, and 90 days postoperatively were 6.3%, 9.6%, and 13.3%, respectively, which were significantly higher than those of the MO (2.7%, 3.6%, 6.2%) and the YO groups (1.2%, 1.8%, 2.4%) (*P* < 0.001).

**Conclusions:**

The majority of elderly patients undergoing hip fracture surgery were female, with falls being the predominant cause of injury. Increasing age was correlated with poorer preoperative mobility, a lower prevalence of three or more comorbidities, and a higher likelihood of receiving spinal anesthesia. Lastly, the probability of ICU admission, complications, and mortality rate increased after surgery.

## Introduction

With the intensification of population aging, the incidence of fractures among the elderly is also increasing on a yearly basis [1,2]. Hip fractures are the most prevalent type of fractures in the elderly, especially in women over 65 years old and men over 70 years old, posing a serious threat to the lives and health of the elderly and emerging as a major health problem worldwide [3]. Surgical intervention can alleviate pain, facilitate care, and reduce the risk of long-term bed rest, making it the preferred treatment option for patients with hip fractures [4]. However, the overall functional decline of elderly individuals is often accompanied by multiple comorbidities, resulting in high postoperative disability and mortality rates [3,5]. Age has been established as an independent risk factor for postoperative complications and mortality [6,7]. Over time, the risk of postoperative complications such as delirium, pneumonia, circulation, cerebrovascular disease, and mortality rate correspondingly increase.

Although the population aged 65 years and above is regarded as elderly, the physiological reserve of the body and physical fitness decrease over time, whilst the risk of surgical anesthesia increases, thereby complicating the decision-making process for surgical anesthesia. In recent years, numerous studies have further stratified elderly individuals into young elderly (65–74 years old), middle-aged elderly (75–84 years old), and elderly elderly (≥ 85 years old) [8–10]. Despite several studies investigating elderly hip fractures [11–13], reports comparing the clinical characteristics and outcomes among different age groups in elderly patients with fracture surgery remain limited. In order to comprehensively elucidate the clinical characteristics and outcomes of these patients, develop effective preventive measures, and provide a robust evidence base for perioperative management, the clinical characteristics and postoperative outcomes of 909 elderly patients with hip fractures treated at our center were summarized and analyzed.

## Methods

### Study design and setting

This single-center, retrospective, observational clinical study was conducted at Liaocheng People’s Hospital. A consecutive sampling method was adopted for participant enrollment. All the elderly patients with hip fractures surgery between October 2020 and April 2023 who met the criteria were included. Data was examined and accessed from 6th May 2024–6th Aug 2024 in our study. The protocol was approved by the Ethics Committee of Liaocheng People’s Hospital 2024102). Oral informed consent was obtained from the patients, or for patients who could not provide consent, from their family members.

### Participants

Patients inclusion criteria were as follows: 1) aged ≥ 65 years old; 2) diagnosed with hip fractures, including intertrochanteric fractures, subtrochanteric fractures, femoral neck fractures, femoral head fractures, and acetabular fractures; and 3) underwent surgical intervention.

Patient exclusion criteria were as follows: 1) missing or unavailable medical records; 2) severe composite injury; 3) inability to obtain informed consent from patients or family members.

Referring to the commonly used classification standards for elderly populations in international clinical research in recent years, patients were divided into three groups based on age: young old group (YO group, 65–74 years old), middle old group (MO group, 75–84 years old), and oldest-old group (OO group, ≥ 85 years old).

### Data collection

Patient data during hospitalization were extracted from the electronic medical record information system of Liaocheng People’s Hospital. Information not available from medical records and post-discharge outcomes was gathered through telephone follow-up.

### Observation indicators

Baseline information: including gender, age, American Society of Anesthesiologists (ASA) classification, ability to perform daily living tasks (assessed using metabolic equivalents (METs) and Holden grade, Nottingham hip fracture score (NHFS)).

Preoperative comorbidities: Documentations encompassed the presence of preoperative cognitive function and 5 common internal medicine diseases. Preoperative cognitive impairment included mild cognitive impairment and Alzheimer’s disease (AD). Internal medicine diseases comprised the following: cardiovascular diseases such as angina, atrial fibrillation, valvular heart disease, myocardial infarction, or hypertension; cerebrovascular diseases such as any cerebrovascular events or transient ischemic attacks; respiratory system diseases such as chronic obstructive pulmonary disease or asthma, excluding acute infections; kidney disease, including pre-existing or non-acute renal injury; Diabetes (definitive diagnosis of diabetes).

Fracture types and injury mechanisms: According to the relationship between the fracture line and the joint capsule, fracture types were classified as intertrochanteric fractures, subtrochanteric fractures, femoral neck fractures, femoral head fractures, or acetabular fractures. The cause of injury was divided into two categories: high-energy injuries and fall-related injuries. The former included traffic accidents, heavy object injuries, and high-altitude fall injuries.

Surgical anesthesia information: including surgical timing, surgical method, anesthesia method, and transfer to the ICU post-surgery.

Postoperative complications referred to the complications that arose during hospitalization after surgery, including pulmonary complications, cardiovascular complications, and cerebrovascular complications. Pulmonary complications included pulmonary infection, pulmonary embolism, and respiratory failure; cardiovascular complications included newly diagnosed arrhythmias requiring treatment, acute myocardial infarction, acute heart failure, and sudden cardiac death; cerebrovascular complications were defined as newly occurring strokes.

Postoperative survival: including survival rates at discharge, 1 month after surgery, and 3 months after surgery.

### Statistical analysis

Statistical analyses were conducted using SPSS 25.0 statistical software. Quantitative data that conformed to a normal distribution were expressed by mean ± standard deviation ($\overset{―}{x}$± s), and inter-group comparisons were conducted using one-way analysis of variance. Non-normally distributed econometric data were presented as median and interquartile range, and inter-group comparisons were conducted using Mann Whitney U test. Categorical variables were expressed as frequency (n) and proportion (%), and inter-group comparisons were conducted using the chi-square test or Fisher’s exact probability method. The test level was set at α = 0.05, and *P* < 0.05 was considered statistically significant.

The study protocol was approved by the Ethics Committee of Liaocheng People’s Hospital (2024102). Oral informed consent was obtained from the patients or family members of patients who could not provide consent.

## Results

This study included 909 elderly patients who underwent hip fracture surgery, ranging between 65–103 years in age, with an average age of (78.6 ± 8.4) years. There were 301 males (33.1%) with an average age of (78.2 ± 8.1) years and 608 females (66.9%) with an average age of (78.7 ± 8.5) years. The male-to-female ratio was 1:2.02. Among them, 332 cases were assigned to the YO group (36.5%), 337 cases to the MO group (37.1%), and 240 cases to the OO group (26.4%) (Table 1).

**Table 1:** Basic characteristics of elderly patients undergoing hip fracture surgery in different age groups [n (%)].

Characteristic	Total (n = 909)	YO group (65 ~ 74y, n = 332)	MO group (75 ~ 84y,n = 337)	OO group (≥85y, n = 240)	*P* value
Gender					
Male	301 (33.1)	106 (31.9)	122 (36.2)	73 (30.4)	0.293
Female	608 (66.9)	226 (68.1)	215 (63.8)	167 (69.6)	
Comorbidities					
Cardiovascular disease	565 (62.2)	187 (56.3)	225 (66.8)	153 (63.7)	0.017
Cerebrovascular disease	294 (32.3)	115 (34.6)	119 (35.3)	60 (25.0)	0.018
Chronic lung disease	140 (15.4)	41 (12.3)	56 (16.6)	43 (17.9)	0.139
Diabetes	159 (17.5)	66 (19.9)	58 (17.2)	35 (14.6)	0.253
Chronic kidney disease	12 (1.3)	3 (0.9)	4 (1.2)	5 (2.1)	0.497
Cognitive impairment	185 (20.4)	40 (12.0)	79 (23.4)	66 (27.5)	< 0.001
Number of comorbidities					
None	203 (22.3)	93 (28.0)	59 (17.5)	51 (21.3)	< 0.001
1 ~ 2	590 (64.9)	186 (56.0)	236 (70.0)	168 (70.0)	
≥3	116 (12.8)	53 (16.0)	42 (12.5)	21 (8.8)	
ASA grade					
<Ⅲ	375 (41.3)	206 (62.1)	119 (35.3)	50 (20.8)	< 0.001
≥ Ⅲ	534 (58.7)	126 (38.0)	218 (64.7)	190 (79.2)	
Preinjury activity tolerance					
< 4METs	514 (56.6)	116 (34.9)	196 (58.2)	202 (84.2)	< 0.001
≥ 4METs	395 (43.4)	216 (65.1)	141 (41.8)	38 (15.8)	
Holden grade					
< 3 grade	175 (19.3)	42 (12.7)	57 (16.9)	76 (31.7)	< 0.001
≥ 3 grade	734 (80.7)	290 (87.3)	280 (83.1)	164 (68.3)	
NHFS score					
≤ 4	500 (55.0)	236 (71.1)	200 (59.4)	64 (26.7)	< 0.001
≥ 5	409 (45.0)	96 (28.9)	137 (40.6)	176 (73.3)	

Note: YO group: patients aged 65–74; MO group: patients aged 75–84; OO group: patients aged 85 and over. ASA: American society of anesthesiologists, MET: metabolic equivalent. NHFS: Nottingham hip fracture score.

### General information comparison

Gender proportion: Across all age groups, the proportion of women was numerically higher than that of men, with no statistically significant differences in gender across age groups (*P* = 0.293). As age increased, preoperative activity tolerance significantly decreased (*P* < 0.05), whereas the proportion of patients with < 4METs significantly increased. Specifically, 84.2% of patients in the OO group had a preoperative activity tolerance of < 4METs, significantly higher than that in the MO (58.2%) and YO groups (34.9%). As anticipated, the proportion of patients with ASA ≥ III significantly increased with increasing age (*P* < 0.001), with the OO group reaching 79.2%, which was significantly higher than the YO (38.0%) and MO groups (64.7%).There were statistically significant differences in the distribution of NHFS scores among patients in different age groups (**P* *< 0.001), and the scores showed a significant increasing trend with advancing age. Specifically, the proportion of patients with an NHFS score ≥5 in the OO group was as high as 73.3%, which was significantly higher than that in the MO group (40.6%) and YO group (28.9%). This suggests that elderly patients (especially those in the OO group) have a higher level of preoperative hip fracture-related risk (Table 1).

Among 909 patients who underwent hip fracture surgery, 706 cases (77.7%) had comorbidities, with 590 cases (64.9%) having 1–2 comorbidities and 116 cases (12.8%) having 3 or more comorbidities. The proportion of patients with ≥ 3 comorbidities in the YO group, MO group, and OO group was 16.0%, 12.5.6%, and 8.8%, respectively (**P* *< 0.001), with cardiovascular diseases accounting for 62.2% (565/909), followed by cerebrovascular diseases 32.3% (294/909). Moreover, significant differences were noted in the prevalence of comorbid cardiovascular diseases and cerebrovascular diseases among the age groups (**P* *= 0.017, **P* *= 0.018, respectively). In contrast, the prevalence of chronic pulmonary diseases, diabetes, and chronic kidney diseases was comparable across age groups (**P* *= 0.497) (Table 1).

Among the enrolled patients, 185 elderly patients (20.4%) underwent hip fracture surgery with preoperative cognitive impairment. Among them, the OO group had the highest proportion of patients with preoperative cognitive impairment (27.5%, 66/240), followed by the MO group (23.4%, 79/337) and the YO group (12.0%, 40/332). Indeed, differences in preoperative cognitive impairment among the different age groups were statistically significant (**P* *< 0.001) (Table 1).

### Causes of injury and fracture types

Among the causes of injury in elderly patients undergoing hip fracture surgery, 831 cases (91.4%) had a history of falls, whereas high-energy injuries, including car accidents, heavy object injuries, and high-altitude falls, accounted for the remaining 78 cases (8.6%). Importantly, the incidence rate of falls was 95.8% (230/240), 91.4% (308/337), and 88.3% (293/332) in the OO, MO, and YO groups. Finally, differences in the cause of injury were significant among the different age groups (**P* *=* *0.006) (Table 2).

**Table 2:** Fracture status of elderly patients undergoing hip fracture surgery in different age groups [n (%)].

	Total (n = 909)	YO group (65 ~ 74y, n = 332)	MO group (75 ~ 84y, = 337)	OO group (≥85y, = 240)	*P* value
Mode of injury					
Fall	831 (91.4)	293 (88.3)	308 (91.4)	230 (95.8)	0.006
High-energy injury	78 (8.6)	39 (11.7)	29 (8.6)	10 (4.2)	
Type of fracture					
Intertrochanteric	443 (48.7)	128 (38.6)	169 (50.1)	146 (60.8)	< 0.001
Subtrochanteric	16 (1.8)	6 (1.8)	8 (2.4)	2 (0.8)	
Femoral neck	435 (47.9)	187 (56.3)	158 (46.9)	90 (37.5)	
Femoral head	3 (0.3)	1 (0.3)	1 (0.3)	1 (0.4)	
Acetabular	12 (1.3)	10 (3.0)	1 (0.3)	1 (0.4)	

Note: YO group: patients aged 65–74; MO group: patients aged 75–84; OO group: patients aged 85 and over.

Among the 909 elderly patients undergoing hip fracture surgery, the incidence of intertrochanteric fractures and femoral neck fractures was comparable (48.7%, 443/909 and 47.9%, 435/909, respectively), whilst subtrochanteric fractures, femoral head fractures, and acetabular fractures were relatively rare. In terms of age distribution, femoral neck fractures were prevalent in the YO (56.3%, 187/332) and MO groups (50.1%, 169/337), whereas intertrochanteric fractures were more common in the OO group (60.8%, 146/240). Notably, the distribution of fracture types was significantly different among the different age groups (**P* *< 0.001) (Table 2).

### Surgical and anesthesia information

In terms of surgical timing, among the 909 patients, 529 (58.2%) underwent surgery within 48 hours after admission. Among them, 164 cases (68.3%) were in the OO group, 202 cases (60%) were in the MO group, and 163 cases (49.1%) were in the YO group, and between-group differences were statistically significant (**P* *< 0.001) (Table 3).

**Table 3:** Information of surgery and anesthesia [n(%)].

	Total (n = 909)	YO group (65 ~ 74y, n = 332)	MO group (75 ~ 84y,n = 337)	OO group (≥85y, n = 240)	*P* value
Time from admission to surgery					
<48h	529 (58.2)	163 (49.1)	202 (60)	164 (68.3)	< 0.001
≥ 48h	380 (41.8)	169 (50.1)	135 (40)	76 (31.7)	
Type of surgery					
Internal fixation	479 (52.7)	152 (45.8)	178 (52.8)	149 (62.1)	< 0.001
Semi hip replacement	387 (42.6)	145 (43.7)	152 (45.1)	90 (37.5)	
Total hip replacement	43 (4.7)	35 (10.5)	7 (2.1)	1 (0.4)	
Type of anesthesia					
Spinal anesthesia	574 (63.1)	183 (55.1)	202 (59.9)	189 (78.8)	< 0.001
General anesthesia	335 (36.9)	149 (44.9)	135 (40.1)	51 (21.3)	
Perioperative blood transfusions					
Yes	432 (47.5)	112 (33.7)	169 (50.1)	151 (62.9)	< 0.001
No	477 (52.5)	220 (66.3)	168 (49.9)	89 (37.1)	
Transferred to ICU after surgery					
Yes	60 (6.6)	15 (4.5)	21 (6.2)	24 (10)	0.032
No	849 (93.4)	317 (95.5)	316 (93.8)	21 (90)	

Note: YO group: patients aged 65–74; MO group: patients aged 75–84; OO group: patients aged 85 and over.

Surgical methods included internal fixation (52.7%, 479/909), semi-hip replacement (42.6%, 387/909), and total hip replacement (4.7%, 43/909). Among the three age groups, internal fixation was the most common (45.8%, 52.8%, 62.1%), followed by hip replacement surgery (43.7%, 45.1%, 37.5%) and total hip replacement surgery (10.5%, 2.1%, 0.4%). Differences in surgical methods among the different age groups were statistically significant (**P* *< 0.001) (Table 3).

Anesthesia methods comprised spinal anesthesia (63.1%, 574/909) and general anesthesia (36.9%, 335/909). The proportion of patients undergoing spinal anesthesia in the OO group (78.8%, 189/240) was higher than that in the MO (59.9%, 202/337) and the YO groups (55.1%, 183/332), and differences in anesthesia methods among the age groups were statistically significant (**P* *< 0.001) (Table 3).

A total of 432 patients (47.5%) received perioperative blood transfusions. Specifically, the proportion of patients who received perioperative blood transfusions in the three age groups was as follows: OO group (62.9%), MO group (50.1%), and YO group (33.7%). As expected, differences in blood transfusion rates between the groups were statistically significant (**P* *< 0.001) (Table 3).

A total of 60 patients (6.6%) were admitted to the ICU postoperatively. Among the three groups, the OO group had the highest proportion of ICU admissions (10%, 24/240), followed by the MO group (6.2%, 21/337) and the YO group (4.5%, 15/332). Likewise, differences among the three groups in terms of ICU admission rates were statistically significant (**P* *=* *0.032) (Table 3).

### Postoperative complications and survival status

The most common postoperative complications were postoperative delirium (31.1%, 283/909), followed by pulmonary complications (18%, 164/909), cardiovascular complications (7.0%, 64/909), and cerebrovascular complications (4.8%, 44/909). Among them, the incidence of postoperative delirium, pulmonary complications, and cardiovascular complications was highest in the YO group, followed by the MO and OO groups (**P* *< 0.001). The incidence of cerebrovascular complications was highest in the MO group (8.3%, 28/337), which was significantly higher than that in the OO (3.8%, 9/240) and YO groups (2.1%, 7/332) (**P* *= 0.001) (Table 4).

**Table 4:** Postoperative complications and mortality [n(%)].

	Total (n = 909)	YO group (65 ~ 74y, n = 332)	MO group (75 ~ 84y,n = 337)	OO group (≥85y, n = 240)	*P* value
**Postoperative complications**					
Delirium	283 (31.1)	76 (22.9)	111 (32.9)	96 (40.0)	< 0.001
Pulmonary complications	164 (18.0)	40 (12.0)	65 (19.3)	63 (26.3)	< 0.001
Cardiovascular complications	64 (7.0)	10 (3.0)	27 (8.0)	27 (11.3)	< 0.001
Cerebrovascular complications	44 (4.8)	7 (2.1)	28 (8.3)	9 (3.8)	0.001
**Postoperative mortality time points**					
In-hospital	28 (3.1)	4 (1.2)	9 (2.7)	15 (6.3)	0.002
30 days	41 (4.5)	6 (1.8)	12 (3.6)	23 (9.6)	< 0.001
90 days	61 (6.7)	8 (2.4)	21 (6.2)	32 (13.3)	< 0.001

Note: YO group: patients aged 65–74; MO group: patients aged 75–84; OO group: patients aged 85 and over

Among the 909 patients, 28 (3.1%) died in the hospital after surgery, 41 (4.5%) died 30 days after surgery, and 61 (6.7%) died 90 days after surgery. Inter-group comparisons revealed that the mortality rates in the YO group, MO group, and OO group progressively increased over time (**P* *< 0.01). Among them, the in-hospital mortality, postoperative 30-day mortality, and postoperative 90-day mortality rates in the OO group were 6.3%, 9.6%, and 13.3%, respectively, which were significantly higher than those in the MO (2.7%, 3.6%, 6.2%) and YO groups (1.2%, 1.8%, 2.4%) (**P* *< 0.001) (Table 4).

## Discussion

The results of this single-center, retrospective clinical study exposed that among the 909 patients aged over 65 years who underwent hip fracture surgery, the majority were patients aged 65–74 years (36.5%) and 75–84 years (37.1%), with a relatively lower proportion of patients aged over 84 years (26.4%). The male-to-female ratio was 1:2.02, and the proportion of female patients steadily increased with advancing age, consistent with findings from earlier studies [3,5]. This distribution may be ascribed to the decrease in the total number of elderly individuals over 84 years old, the high incidence of endocrine dysfunction and osteoporosis in postmenopausal elderly women, and the higher life expectancy of women compared to men. On average, roughly 58% of patients had ASA ≥ III in this study, slightly higher than 52% in the UK [14], with 79.2% being the elderly group. This suggests that the risk of postoperative complications and mortality increases with age [15].

As is well documented, patients with hip fractures frequently suffer from multiple comorbidities. The NHFS is a widely used scoring system for predicting postoperative mortality in patients with hip fractures and has been validated in multiple studies [16,17]. This study utilized five internal medicine system diseases to access the prevalence of comorbidities among patients. A total of 77.7% (706/909) of patients had comorbidities, with cardiovascular diseases being the most prevalent, in line with the observations of previous studies [18]. The proportion of patients with ≥ 3 comorbidities significantly decreased with age, with the lowest proportion in the OO group (8.8%), possibly due to the close correlation between fewer comorbidities and longevity. Cognitive dysfunction encompasses a spectrum of cognitive impairments that range from mild cognitive impairment to AD. Of note, age has been identified as a significant risk factor for cognitive dysfunction. Herein, the proportion of patients with preoperative cognitive impairment gradually increased with advancing age, consistent with the characteristics of the disease. Importantly, AD is a risk factor for falls, and patients with AD generally receive multiple drugs, thereby increasing, the risk of falls. Therefore, patients with AD are more prone to hip fractures. The incidence of cognitive impairment in this study was 20.4% (185/909), including not only AD patients but also those with mild cognitive impairment, which is lower than the one-third of hip fracture patients with AD recorded in a recent study [19]. This discrepancy may be attributed to the insufficient self-awareness of their own conditions among AD patients.

Intertrochanteric fractures and femoral neck fractures accounted for the majority of hip fractures, with similar incidence rates of 47.7% (443/909) and 47.9% (435/909), respectively. In the three age groups, the proportion of patients with intertrochanteric fractures increased with advancing age, whereas the proportion of those with femoral neck fractures decreased. Previous studies have concluded that patients with intertrochanteric fractures are generally older, with lower bone density, and poor health status, all of which have been identified as independent predictors of intertrochanteric fractures [20]. This is consistent with the increasing proportion of intertrochanteric fractures in the age group in this study. This study demonstrates that falls constitute the primary etiology of hip fractures in elderly populations, accounting for 91.4% of cases, with the proportion of fall-related injuries exhibiting a positive correlation with advancing age (95.8% in the HO group versus 91.4% in the MO group versus 88.3% in the YO group). These findings underscore the imperative for developing evidence-based fall prevention strategies. Considering the demographic characteristics of the study cohort and current clinical guidelines, fall prevention initiatives should address the following key factors: The increased bone fragility associated with osteoporosis in elderly individuals predisposes them to fractures from minimal trauma [21]. Age-related decline in postural stability and coordination, coupled with the high prevalence of comorbidities and polypharmacy, further exacerbates fall risk. Consequently, the implementation of targeted, multi-factorial interventions is essential. Current evidence supports the following strategies: (1) Comprehensive fall-risk assessment through routine screening for balance impairment, polypharmacy, and environmental hazards to identify high-risk individuals; (2) Implementation of geriatric co-management models, particularly orthogeriatric approaches, which integrate osteoporosis management, mobility rehabilitation, and coordinated care to optimize outcomes; (3) Post-discharge rehabilitation programs, including telerehabilitation and supervised exercise regimens, which have demonstrated efficacy in reducing recurrent falls and enhancing functional recovery. Future research and clinical implementation should focus on developing scalable, guideline-concordant interventions to address existing gaps in treatment adherence and accessibility.

Early surgical treatment can alleviate patient pain and minimize the risk of postoperative complications and mortality. Guidelines recommend surgery to be performed within 48 hours after admission. Herein, 68.3% of elderly patients underwent surgery within 48 hours after admission. A common reason for delayed surgery is the high perioperative risk associated with comorbidities in patients, which requires further adjustment and treatment before surgery. In terms of anesthesia methods, especially for elderly patients, our center prioritized spinal anesthesia in patients without contraindications, accounting for the higher proportion of patients receiving intraspinal anesthesia (63.1%) in this study compared to general anesthesia (36.9%). To manage positional changes and postoperative pain, all patients underwent ultrasound-guided iliac fascia block pre-operatively, a procedure whose clinical efficacy has been validated in several clinical studies. Our center adjusted the transfusion thresholds for asymptomatic patients to Hb < 90g/L and for symptomatic patients to Hb < 100g/L. Patients with unstable postoperative vital signs were transferred to the ICU. On average, nearly half (47.5%) of patients received blood transfusions during the perioperative period, and 6.6% of patients were transferred to the ICU after surgery. As age increased, the proportion of patients requiring blood transfusions and transfer to the ICU significantly increased. It may be due to the decline in nutritional status, osteoporosis, and anticoagulant therapy in elderly patients, suggesting an elevated perioperative risk in this population.

The most common postoperative complications in this study were postoperative delirium, pulmonary complications, cardiovascular complications, and cerebrovascular complications. Among them, cardiovascular disease was ranked highest, and cardiovascular events are the main cause of postoperative death in patients with hip fracture [22]. Therefore, enhancing perioperative management for high-risk cardiac patients holds significations in improving the prognosis of hip fracture patients, including maintaining ideal hemodynamic status during surgery, prophylactic administration of vasoactive drugs to maintain blood pressure not lower than 10% of preoperative baseline blood pressure, and preventing the occurrence of cardiovascular events [18]. According to earlier studies, the in-hospital mortality rate of elderly patients with hip fractures ranges from 2.3% to 13.9% and increase within 6 months [5]. In the present study, the average in-hospital mortality rate was 3.1% and was higher at 30 and 90 days after surgery. The in-hospital mortality rate of elderly patients is as high as 6.3%, while the 90-day postoperative mortality rate is 13.3%, highlighting the need to consider age when evaluating the risk of postoperative mortality.

The limitations of this study were as follows: 1) Only elderly patients with hip fractures who underwent surgical treatment were included, whereas those who underwent conservative treatment were excluded. 2) This study only analyzed postoperative complications and mortality, without evaluating postoperative functional outcomes (e.g., mobility, independence, quality of life). This makes it impossible to fully assess the “actual benefits” of surgery for different age groups, analyze the association between age and the speed/degree of functional recovery, or develop accurate age-specific postoperative rehabilitation plans. 3) Despite using consecutive sampling to include all eligible patients at our single center, regional differences in patient demographics (e.g., baseline comorbidities, access to care) and potential underrecording of certain preoperative conditions (e.g., mild cognitive impairment) may have introduced subtle selection biases. Further multicenter studies with larger samples are warranted to validate our findings.

## Conclusion

The majority of elderly patients undergoing hip fracture surgery were female, with falls being the predominant cause of injury. Increasing age was correlated with poorer preoperative mobility, a lower prevalence of three or more comorbidities, and a higher likelihood of receiving spinal anesthesia. Lastly, the probability of ICU admission, complications, and mortality rate increased after surgery.

## Supporting information

S1 Data Raw data. (XLSX)
